# Prevalence and Correlates of Hepatitis B in Pregnancy: Evidence From a Cross‐Sectional Study in Bono East Region, Ghana

**DOI:** 10.1002/hsr2.71263

**Published:** 2025-09-18

**Authors:** Dennis Bardoe, Daniel Hayford, Robert Bagngmen Bio, Denis Dekugmen Yar

**Affiliations:** ^1^ Department of Public Health Education Akenten Appiah‐Menka University of Skills Training and Entrepreneurial Development Mampong Ghana; ^2^ Department of Integrated Science Education Akenten Appiah‐Menka University of Skills Training and Entrepreneurial Development Mampong Ghana; ^3^ Department of Disease Control and Epidemiology College of Health and Well‐Being Kintampo Ghana

**Keywords:** Bono East Region, determinants, epidemiology, hepatitis B virus, horizontal transmission, maternal health, pregnancy, seroprevalence, vaccination coverage, vertical transmission

## Abstract

**Background and Aims:**

Hepatitis B virus (HBV) infection during pregnancy is associated with adverse maternal and neonatal outcomes. Although Ghana's national HBV prevalence is 8.36%, regional disparities are often underexplored. The Bono East Region, characterized by its trade‐driven labor force, population mobility, and limited maternal health access, may experience different HBV transmission patterns. In response, our study quantified HBV prevalence and identified its correlates among pregnant women in the Bono East Region.

**Methods:**

This study employed a cross‐sectional design with a mixed‐methods approach among 1430 pregnant women. Data were collected using serological tests, closed‐ended questionnaires, in‐depth interviews, and focus group discussions. Quantitative data were analyzed using descriptive statistics, chi‐square tests, and logistic regression (bivariate at *p* ≤ 0.25, and multivariate at *p* < 0.05 with 95% confidence intervals). Thematic analysis of the qualitative data was conducted following a four‐stage interpretive framework.

**Results:**

The study found an HBV prevalence of 1.82% (95% CI: 1.24–2.65). Several factors were independently associated with increased odds of infection. These included sharing sharp items (AOR = 3.24; 95% CI: 2.98–5.71), unprotected sex (AOR = 5.04; 95% CI: 1.98–6.81), unsafe abortion (AOR = 3.51; 95% CI: 1.16–5.53), blood transfusion (AOR = 6.82; 95% CI: 2.53–8.34), body piercing (AOR = 2.50; 95% CI: 1.85–4.31), street nail trimming (AOR = 3.63; 95% CI: 1.37–5.62), being unmarried (AOR = 17.51; 95% CI: 5.47–25.98), low income (AOR = 6.28; 95% CI: 1.58–11.94), living in compound houses (AOR = 7.25; 95% CI: 1.23–12.43), secundigravidae (AOR = 5.37; 95% CI: 2.45–11.80), and blood group O (AOR = 4.36; 95% CI: 1.40–6.54).

**Conclusion:**

The HBV prevalence was lower than the national average of 8.36%. However, the associated determinants highlight the need for expanded vaccination coverage and improved health education.

## 1 Introduction

Advances in medical practices and research have positively strengthened the well‐being of people globally. Despite these improvements, the incidence of infectious diseases is widespread, affecting vulnerable people, including pregnant women [1]. The (re)emergence of these diseases, particularly in sub‐Saharan Africa, could be attributed to a lack of awareness, mother‐to‐child transmission, horizontal transmission in early childhood, poor disease surveillance, and broken healthcare systems [2]. In response, the United Nations pledged in Sustainable Development Goal 3 (SDG 3.3) to end the epidemics of AIDS, tuberculosis, malaria, hepatitis, various water‐borne diseases, neglected tropical diseases, and non‐communicable diseases by 2030 [3]. Some of the core strategies that are being implemented to help achieve this include the intensification of awareness, the right diagnoses and treatment, and effective immunization programmes [4]. These strategies are estimated to reduce the burden of infections by 65%, especially in countries with moderate to high endemicity [5]. Despite these interventions and some moderate gains, hepatitis B virus (HBV) infection in pregnancy remains endemic in most sub‐Saharan African countries [6, 7].

Globally, over 254 million people were chronically infected with HBV in 2022, resulting in 1.1 million deaths [7]. HBV prevalence remains particularly high in the Western Pacific and African regions, as highlighted in regional surveillance reports. Specifically, 97 million and 65 million people with chronic HBV dwell in these regions, respectively [7]. Ghana's national prevalence of HBV was between 8.36% and 8.6% in 2020, with a death toll of 3118 [8]. In settings where the disease is highly prevalent, infants are commonly exposed through perinatal transmission or early contact with contaminated blood [7]. Transmission could also occur through certain lifestyle practices, such as unprotected sexual activities, and other risk factors, including unsafe blood transfusions, needlestick injuries, tattooing, piercing, unsterile medical equipment, and contact with infected bodily fluids [7]. Evidence suggests that HBV infection in pregnant women may contribute to a range of adverse maternal and neonatal outcomes, including preterm birth, hypertensive complications, metabolic disorders, and fetal or neonatal death [7].

The epidemiological trends, transmission dynamics, and pregnancy‐related complications highlight the burden of this disease and could intensify given the absence of country‐ and region‐based empirical data. Although previous national surveys have reported a higher prevalence, regional disparities in health outcomes are common and often shaped by complex, context‐specific factors. Investigating these patterns at the subnational level is essential not only for identifying regions with disproportionately high burdens but also for understanding areas with lower‐than‐expected prevalence, which may reveal protective factors or differences in access, awareness, or behavior.

Compared to national estimates of 17 and 28 deaths per 1000 live births, the Bono East Region recorded significantly higher mortality rates for both neonates and infants (24 and 36 deaths per 1000 live births, respectively) [9]. This remained a critical burden and could be attributed to numerous factors, including inadequate access to maternal healthcare and the persistence of infectious diseases like HBV [9]. Besides, distinct socioeconomic conditions within the region may shape both the distribution and intensity of HBV infection among the population [6]. As the region in the heart of the country, agriculture and retail stand to be the predominant labor force. Given these throughout‐the‐year economic activities, a workforce from all parts of the country temporarily travels to the regions to work for income [4]. This seasonal influx of workers from various regions of the country increases population mobility, which has been identified as a significant driver of infectious diseases, including HBV [10]. Furthermore, temporary accommodations could lead to overcrowding in households and facilitate shared living conditions in which communal practices such as sharing personal items become more common. These practices increase the risk of horizontal transmission of HBV [7] and, hence, have enormous public health implications, as emergencies that occur anywhere in the country or the sub‐region could spread to the region and vice versa [11].

Available literature on HBV infection during pregnancy remains limited within the Bono East Region of Ghana. Before our study, the only peer‐reviewed study reported a seroprevalence of 9.6% among women in the Kintampo Municipality [12]. This limited availability of regional epidemiological evidence has made it difficult to formulate data‐driven policies to improve maternal and neonatal health outcomes. This study offers new evidence on HBV prevalence and its drivers among pregnant women to guide context‐specific public health responses and policy development.

## 2 Materials and Methods

### 2.1 Study Design

A multicentre hospital‐based mixed‐method cross‐sectional study was conducted from January 22 to April 15, 2024.

### 2.2 Study Area

**Figure 1:**
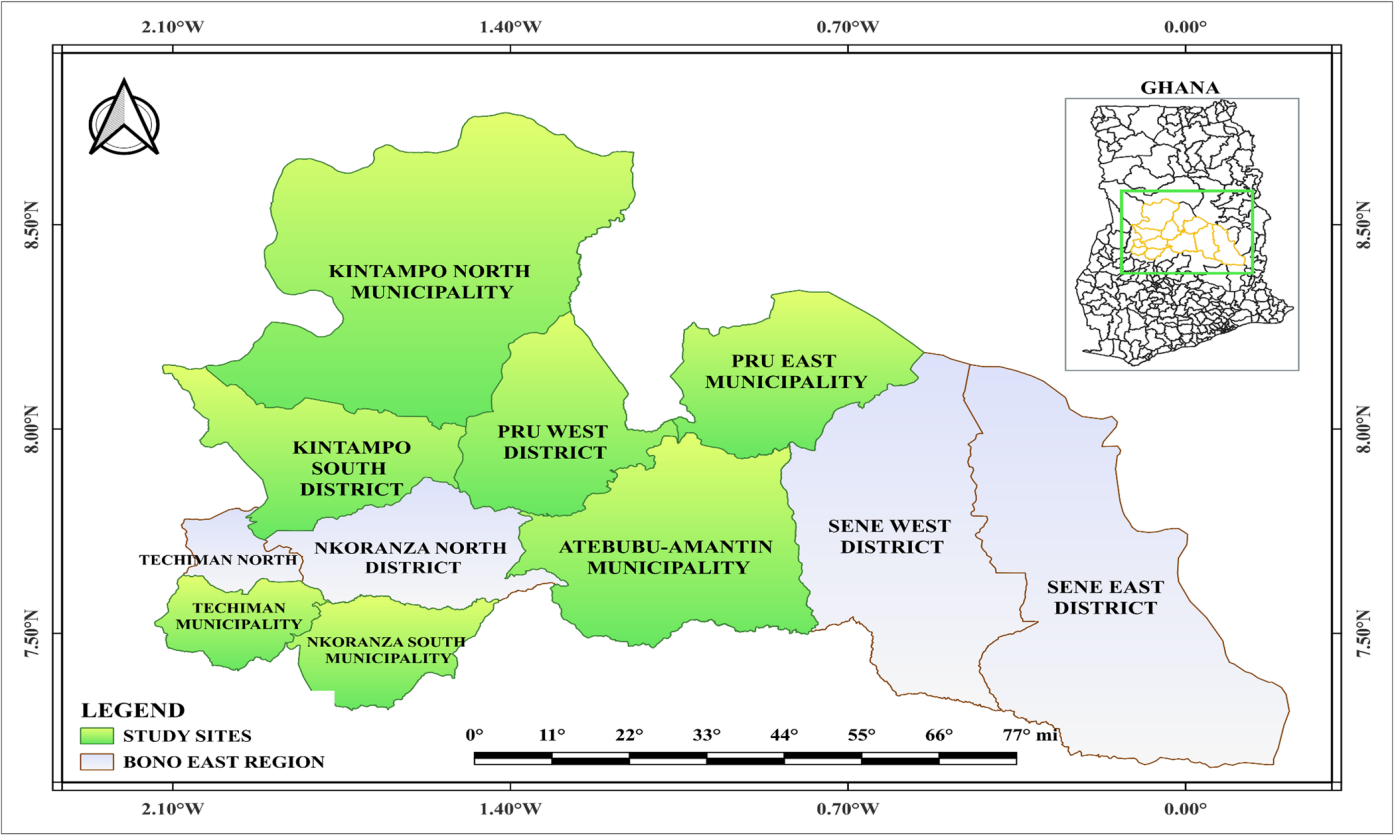
Map of the study sites, designed with QGIS. Basemap shapefiles are accessible. www.geoboundaries.org/countryDownloads.html.

The study was conducted across selected health facilities in seven municipalities and districts within Ghana's Bono East Region (Figure 1). This region, located in central Ghana, spans an estimated 22,952 km^2^. It has a projected population of around 1.2 million, yielding a population density of approximately 49 people per square kilometer [13]. The region shares borders with the Savannah Region to the north, the Bono Region to the west, the Ashanti Region to the south, and the Volta Lake to the east [13]. The health infrastructure comprises over 360 facilities, among them district hospitals, general hospitals, health centers, maternity homes, private clinics, and over 280 active Community‐based Health Planning and Services zones, which predominantly offer secondary‐level care [13].

### 2.3 Study Population

Pregnant women who sought ANC services at seven healthcare facilities were enrolled in the study.

### 2.4 Sample Size Estimation

To estimate the required number of participants, we used Slovin's formula [14].

$$n=\frac{N}{1+N{\left(e\right)}^{2}}.$$

In this context, (*N*) refers to the 2022 estimated population of pregnant women within the study areas, recorded as 33,395. To minimize sampling variability and improve inferential accuracy, a 3% margin of error (*e*) was incorporated in determining the required sample size [15].

$$=\frac{33395}{1+{33395(0.03)}^{2}},\approx 1075.$$

To account for potential nonresponse, a 35% adjustment was applied to the initial estimate of 1075 participants, resulting in a final target sample size of 1452. This adjustment was intended to enhance the reliability of the findings. The sample was then distributed across selected health facilities proportionally, based on the population representation of each district.

### 2.5 Sampling Procedure

This study employed a multistage sampling approach. In the initial stage, five municipalities and two districts were selected based on convenience. Subsequently, the main health facilities located in the capitals of these municipalities and districts were purposively chosen. This was because they function as the primary referral centers with high patient attendance, thus offering access to a broad patient population and relevant clinical records. At each selected facility, pregnant women were enrolled through systematic random sampling. Recruitment was carried out by selecting every third attendee, beginning at a randomly determined position in the queue. The starting point was selected manually by randomly drawing one folded slip from three numbered (1 to 3) papers, and the number drawn determined the first participant in the sequence. This process continued until the target sample size was met.

### 2.6 Inclusion and Exclusion Criteria

Pregnant women were eligible for inclusion if they resided in the selected municipality or district, were receiving antenatal care (ANC) services at the participating health facilities, and provided informed consent. Participants were excluded if they experienced any form of discomfort during the data collection process. In addition, where effective communication could not be ensured due to language barriers and no interpreter was available, such individuals were respectfully withdrawn despite prior consent.

### 2.7 Recruitment of Participants

**Figure 2:**
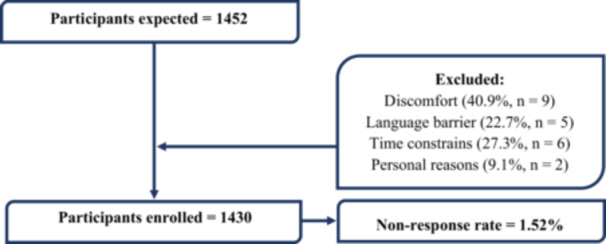
Flow diagram showing the recruitment process and the nonresponse rate.

For quantitative data, 1452 pregnant women were recruited during their routine ANC visits from December 13, 2023, to January 18, 2024. Following a preliminary eligibility assessment, participants who qualified were given full study information and asked to give informed consent in accordance with ethical guidelines. Twenty‐two of the pregnant women did not participate for various reasons (Figure 2). For qualitative data, 10 pregnant women and 1 senior midwife from each health facility were included. Pregnant women were selected based on their experiences with maternal healthcare services (including attending ANC sessions, undergoing routine health screenings and check‐ups, and receiving health education on pregnancy‐related complications) to enhance free and open discussions. The senior midwives, by contrast, were not participants in the study but were engaged as key informants to provide context and professional insights that would enrich our understanding of the quantitative findings. Their perspectives were particularly valuable in interpreting the maternal health service experiences reported by pregnant women, given their extensive frontline involvement in service delivery.

### 2.8 Data Collection Methods

#### 2.8.1 Quantitative Data

Obstetric information was extracted from ANC logbooks at the health facilities. To assess sociodemographic profiles and behavioral risk factors, we administered a structured, closed‐ended questionnaire. The questionnaire was developed with reference to tools used in the Ghana Demographic and Health Survey, and modified to suit the specific objectives of this study [9]. Content validation was undertaken by consulting academic professionals with expertise in epidemiology.

#### 2.8.2 Blood Sample Collection and Serological Examination

A serological examination was performed to determine the proportion of pregnant women who were and were not infected. At the laboratory, the antecubital fossa (inner bend of the left elbow) of the pregnant woman was cleaned with 70% denatured alcohol. A trained medical laboratory technician collected approximately 5 mL of venous blood from the median cubital vein into an EDTA (purple‐top) tube. The samples were allowed to stand at ambient temperature for 30 min to facilitate clotting, after which they were centrifuged at 1500 rpm for 3 min to obtain serum. Using the Onsite HBsAg Combo Rapid Test Kit (CTK Biotech, San Diego, USA), two to three drops of serum were placed onto the serum pad, followed by the addition of an equal amount of buffer solution. The kit reports a sensitivity of 100% and a specificity of 98.3% (95% CI: 95.2–99.4) [16]. Results were interpreted within 5–10 min. To ensure test accuracy, internal quality control was performed using stored hepatitis B surface antigen (HBsAg)‐positive serum samples. All positive results were repeated to rule out potential false positives from defective kits. A test was considered reactive when pink lines appeared at both the control (**C**) and test (**T**) zones; nonreactive when only the control (**C**) line was visible; and invalid when no line or only the test (**T**) line appeared. Invalid tests were retested for confirmation [16].

#### 2.8.3 Qualitative Data

To complement the quantitative findings, this study engaged pregnant women in seven focus group discussions (FGDs) and conducted in‐depth interviews (IDIs) with senior midwives from each of the participating health facilities.

### 2.9 Data Management and Analysis

Before analysis, the data were reviewed to confirm completeness, internal consistency, and interpretability as part of the data management process.

### 2.10 Pre‐Specified Analyses

To determine HBV prevalence and investigate potential predictors, the study utilized a predefined approach comprising descriptive summaries, chi‐square analyses, and logistic regression models (bivariate and multivariate) to explore predictors of HBV infection in the study population.

### 2.11 Exploratory Analyses

This study also explored patterns such as municipality or district differences in HBV prevalence and variations linked to obstetric characteristics. In addition, insights from qualitative data gathered through FGDs and IDIs were thematically analyzed to interpret unexpected patterns emerging from the quantitative findings.

### 2.12 Statistical Tests and Significance Thresholds

We used descriptive statistics to summarize the data, including frequencies, percentages, and means with standard deviations for continuous variables. Chi‐square (*χ*²) tests for homogeneity of proportions were used to examine differences in proportions across categorical variables. Initial associations were assessed using bivariate logistic regression (**Model I**), and variables with *p*‐values ≤ 0.25 were retained for inclusion in the multivariable logistic regression analysis (**Model II**). Crude odds ratios were reported for the bivariate analyses, and adjusted odds ratios (AORs) with 95% confidence intervals (CIs) were reported for the multivariate analysis. A *p*‐value < 0.05 was considered statistically significant for the multivariate model (**Model II**). All statistical tests were two‐tailed.

To assess the assumption of independence among residuals, we applied the Durbin–Watson statistic. A result of 1.970 indicated no evidence of autocorrelation, as it falls within the acceptable range (1.5–2.5). We also evaluated multicollinearity among explanatory variables using variance inflation factors, ensuring no collinearity issues were present. Analyses were conducted using STATA version 17 (StataCorp LLC, College Station, TX, USA), with interpretation aligned with accepted reporting standards to ensure clarity of effect estimates and their precision.

For qualitative data, we adopted an inductive thematic analysis approach guided by four analytic steps: transcription, profiling, coding, and theme development. Audio recordings from FGDs and IDIs were transcribed into English and checked for accuracy against the recordings. Each transcript was assigned a code reflecting the study location. FGDs and IDIs were labeled by district or municipality: (FGD‐ATBM, FGD‐KSD, FGD‐KNM, FGD‐NSM, FGD‐TM, FGD‐PWD, and FGD‐PED) and (IDI‐ATBM, IDI‐KSD, IDI‐KNM, IDI‐NSM, IDI‐TM, IDI‐PWD, and IDI‐PED). Manual coding was used to identify emerging patterns and key themes relevant to the study objectives.

### 2.13 Ethical Approval and Consent for Participation

Ethical clearance for this study was granted by the Committee on Human Research, Publication, and Ethics (CHRPE) at the School of Medical Sciences, Kwame Nkrumah University of Science and Technology (Approval no. CHRPE/AP/1081/23; dated December 11, 2023), in accordance with the Declaration of Helsinki. Before participation, all individuals provided written informed consent either by signature or thumbprint after the study was fully explained to them. For participants under the age of 18, additional consent was obtained from a parent or legal guardian.

## 3 Results

### 3.1 Baseline Characteristics of Pregnant Women

Table 1 summarizes the participants' sociodemographic and obstetric characteristics. The women were aged 17–40 years (mean: 28.8  ±  3.73; 95% CI: 28.63–29.02), with 76.1% falling within the 26–40 age group. Most participants were married (91.7%), had some formal education (71.1%), and identified as Christian (83.5%). Nearly all were employed (97.6%) across occupations such as trading (42.9%), hairdressing (19.2%), seamstressing (16.4%), farming (14.3%), domestic work (3.1%), and the civil service (1.8%). In terms of income, 48.4% reported earnings between Ghȼ 100 and 500. Extended family living arrangements were common (75.9%), with 70.6% residing in compound houses, and 89.1% in block structures with iron sheet roofing. Household sizes of one to five persons accounted for 62.6% of participants. Regarding obstetric history, 43.6% were secundigravida, and 79.3% were multiparous. ANC attendance ranged from one to nine visits (mean: 2.85  ±  1.9; 95% CI: 2.76–2.95), with most (69.3%) attending one to three times. At the time of the study, 73.5% were in their first trimester. For clinical characteristics, the majority (95.9%) had no G6PD deficiency, 96.9% tested negative for sickling, and 29.5% had blood group A.

**Table 1:** Baseline characteristics of pregnant women.

Variable	*n*	%
Age (mean ± SD)	28.8 ± 3.73 (95% CI: 28.63–29.02)	
18–25	174	12.2
26–30	1088	76.1
31–40	168	11.7
Marital status		
Not married	118	8.3
Married	1312	91.7
Educational attainment		
No formal education	414	28.9
Formal education	1016	71.1
Religious affiliation		
Islam	233	16.3
Christianity	1194	83.5
African tradition	3	0.2
Labor force		
Employed	1396	97.6
Unemployed	34	2.4
Type of occupation		
Hairdressing	275	19.2
Seamstress	234	16.4
Farming	204	14.3
Civil service	26	1.8
Trading/marketing	613	42.8
Domestic activities	44	3.1
Unemployed	34	2.4
Monthly income		
None	34	2.4
Ghȼ 100–500	692	48.4
Ghȼ 600–1000	538	37.6
Above Ghȼ 1000	166	11.6
Household structure		
Extended	1086	75.9
Nuclear	344	24.1
Household type		
Compound house	1009	70.6
Self‐contain house	421	29.4
Household category		
Mud with thatch	27	1.9
Mud with iron sheets	129	9
Blocks with iron sheets	1274	89.1
Number of people in a household		
1–5	895	62.6
6–10	532	37.2
11–15	3	0.2
Gravidity		
Primigravida	335	23.4
Secundigravida	624	43.6
Multigravida	471	32.9
Parity		
Nulliparous	296	20.7
Multiparous	1134	79.3
ANC visits (mean ± SD)	2.85 ± 1.9 (95% CI: 2.76–2.95)	
1–3	990	69.3
4–6	340	23.8
7–8	100	7.0
Gestation age		
First trimester	1051	73.5
Second trimester	256	17.9
Third trimester	123	8.6
G6PD		
No defect	1371	95.9
Partial defect	49	3.4
Full defect	10	0.7
Blood group		
O	337	23.6
A	422	29.5
B	420	29.4
AB	251	17.6
Sickling		
Positive	45	3.1
Negative	1385	96.9

Abbreviations: %, percentage; ANC, antenatal care; G6PD, glucose‐6‐phosphate dehydrogenase; *n*, frequency.

### 3.2 Prevalence of HBV Infection

As shown in Table 2, 1.82% (95% CI: 1.24–2.65) of pregnant women tested positive for HBV infection [15]. The difference in HBV seropositivity between infected and uninfected individuals was statistically significant (*χ* ^2^ = 1327.891, DF = 1, *p* < 0.05). Among the study areas, Nkoranza South Municipality reported the highest prevalence (2.96%), while the lowest was observed in Kintampo South District (1.06%).

The prevalence was reinforced by feedback during the IDIs. These key informants noted that:

“…Pregnancy inherently brings various medical complications. In my experience in this facility, the most common medical complications include UTIs, malaria, HBV, HIV, and gestational diabetes. Among these, malaria, UTIs, and HBV emerge as the most prevalent…”

(Senior Midwives, IDI‐TM, IDI‐PED, and IDI‐ATBM)

“…For now, I can mention malaria, UTIs, and upper respiratory tract infections as the most prevalent diseases. Even though HBV is also a concern, it is not as common as the other three mentioned…”

(Senior Midwife, IDI‐KSD)

**Table 2:** Prevalence of HBV infection among pregnant women.

	HBV status			
Municipality	Positive (*n*)	Negative (*n*)	Sample size (*n*)	Prevalence (%)
Atebubu‐Amantin Municipal	3	220	223	1.35
Kintampo North Municipal	4	261	265	1.51
Kintampo South District	1	93	94	1.06
Nkoranza South Municipal	5	164	169	2.96
Techiman Municipal	7	392	399	1.75
Pru East District	4	173	177	2.26
Pru West District	2	101	103	1.94
Total	26	1404	1430	1.82

Abbreviations: %, percentage; *n*, Frequency.

### 3.3 Behavioral Determinants of HBV Infection

The behavioral determinants of HBV infection among pregnant women are summarized in Table 3. The logistic regression model demonstrated a good fit and was statistically significant, explaining approximately 62% of the variability in HBV status [LR *χ*² (10) = 83.51, Prob > *χ*² < 0.001, Log‐likelihood = −88.19, Pseudo *R*² = 0.6213]. During the multivariate analysis, six factors remained significantly associated with HBV infection. Specifically, the odds of HBV were 3.24 times higher among pregnant women with a long‐standing tradition of communal sharing of sharp objects with other persons compared to those who did not share sharp objects (AOR = 3.24; 95% CI: 2.98–5.71; *p* = 0.035). Similarly, pregnant women who engaged in unprotected sex had 5.04 times greater odds of infection compared with those who did not (AOR = 5.04; 95% CI: 1.98–6.81; *p* < 0.001). Likewise, pregnant women with a history of unsafe abortion had 3.51 times higher odds of infection relative to those without such a history (AOR = 3.51; 95% CI: 1.16–5.53; *p* = 0.025). Furthermore, the odds of HBV infection were found to be 6.82 times higher in pregnant women who had previously received blood transfusions compared to those who had not (AOR = 6.82; 95% CI: 2.53–8.34; *p* < 0.001). Moreover, pregnant women who underwent body piercing in nonclinical settings had 2.50 times higher odds of infection than those who did not (AOR = 2.50; 95% CI: 1.85–4.31; *p* = 0.029). Lastly, pregnant women who reported using informal personal care services, such as nail trimming performed by street providers, were more likely to test positive for HBV compared to those who did not use such services (AOR = 3.63; 95% CI: 1.37–5.62; *p* = 0.009).

**Table 3:** Behavioral determinants of HBV infection among pregnant women.

		HBV status					
	Frequency	Positive	Negative	Model I		Model II	
Variable	*n* (%)	*n* (%)	*n* (%)	COR (95% CI)	*p* value	AOR (95% CI)	*p* value
Reproductive tract infection (RTI)							
Yes	1320 (92.3)	21 (1.5)	1299 (90.8)	0.33 (0.12–0.91)	0.033*	0.52 (0.13–1.96)	0.337
No	110 (7.7)	5 (0.3)	105 (7.3)	1		1	
Sharing a sharp object							
Yes	205 (14.3)	12 (0.8)	193 (13.5)	5.37 (2.45–7.80)	< 0.001*	3.24 (2.98–5.71)	0.035*
No	1225 (85.7)	14 (1.0)	1211 (84.7)	1		1	
Unprotected sex							
Yes	137 (9.6)	11 (0.8)	126 (8.8)	4.43 (3.34–6.54)	< 0.001*	5.04 (1.98–6.81)	< 0.001*
No	1293 (90.4)	15 (1.0)	1278 (89.4)	1		1	
Unsafe abortion							
Yes	78 (5.5)	10 (0.7)	68 (4.8)	12.27 (5.37–18.07)	< 0.001*	3.51 (1.16–5.53)	0.025*
No	1352 (94.5)	16 (1.1)	1336 (93.4)	1		1	
Blood transfusion							
Yes	232 (16.3)	17 (1.2)	215 (15.0)	10.44 (4.49–13.73)	< 0.001*	6.82 (2.53–8.34)	< 0.001*
No	1198 (83.8)	9 (0.6)	1189 (83.1)	1		1	
Body piercing in a nonclinical setting							
Yes	192 (13.4)	9 (0.6)	183 (12.8)	3.53 (1.55–4.04)	0.003*	2.50 (1.85–4.31)	0.029*
No	1238 (86.6)	17 (1.2)	1221 (85.40	1		1	
Tattooing							
Yes	43 (3.0)	1 (0.1)	42 (2.9)	1.29 (0.17–9.80)	0.101	1.19 (0.11–12.5)	0.180
No	1387 (97.0)	25 (1.7)	1362 (95.2)	1		1	
Street nail trimming							
Yes	180 (12.60	9 (0.6)	171 (12.0)	3.81 (1.67–8.69)	< 0.001*	3.63 (1.37–5.62)	0.009*
No	1250 (87.4)	17 (1.2)	1233 (86.2)	1		1	
Uptake of alcoholic beverages							
Yes	114 (8.0)	6 (0.4)	108 (7.6)	3.6 (1.41–5.15)	0.007*	1.27 (0.36–4.41)	0.210
No	1316 (92.0)	20 (1.4)	1296 (90.6)	1		1	
HBV vaccination							
Yes	367 (25.7)	12 (0.8)	355 (24.8)	0.39 (0.18–0.86)	0.002*	0.93 (0.34–2.48)	0.190
No	1063 (74.3)	14 (1.0)	1049 (73.4)	1		1	

Abbreviations: %, percentage; AOR, adjusted odds ratio; COR, crude odds ratio; HBV, hepatitis B virus; *n*, frequency; RTI, reproductive tract infection.

* *p* < 0.05.

From the FGDs, the lifestyles of some pregnant women substantiated the identified risk factors for HBV. Some pregnant women remarked:

“…I heard about this illness at secondary school. I know it can be transmitted through blood transfusions…”

(1st Pregnant woman, FGD‐PED)

“…As for me, I live with my family members in a household where everything is shared. This sharing tradition has been part of our household for as long as I can remember…”

(6th Pregnant woman, FGD‐KSD)

“…The abundance of earrings you see adorning my ears and nose is a distinct marker of my tribe's identity. In our culture, the more earrings one has, the more beautiful and esteemed they are. We have individuals whom our grandmothers have specifically trained to pierce us whenever we desire…”

(3rd Pregnant woman, FGD‐TM)

“…When I became pregnant for the third time, the nurses at the ANC advised us to undergo vaccination. However, I could not go, and since then, I have been hesitant. I do wish I could go for the vaccination, but I have some doubts lingering in my mind…”

(4th Pregnant woman, FGD‐NSM)

“…For me, I mostly rely on the services of street nail trimmers (Abookyi) for my nail care, even before I became pregnant. Now that I am pregnant, I still call them because I cannot bend comfortably to trim my nails. Also, they do a great job, and my nails look clean and well‐groomed afterwards…”

(7th Pregnant woman, FGD‐ATM)

### 3.4 Sociodemographic and Obstetric Factors Associated With HBV Infection

The sociodemographic and obstetric predictors of HBV infection are summarized in Table 4. The logistic regression model incorporating 12 predictors was statistically significant. The Pseudo *R* ^2^ of 0.6487 indicates that the model explains approximately 65% of the variability in the outcome (HBV), suggesting a strong model fit [LR *χ* ^2^ (12) = 141.20, Prob > *χ*² < 0.0000, Log‐likelihood = −58.072, Pseudo *R* ^2^ = 0.6487]. During the multivariate analyses, pregnant women who were not married had significantly higher odds of HBV infection compared with married women (AOR = 17.51; 95% CI: 5.47–25.98; *p* < 0.001). In terms of income, women earning Ghȼ 100–500 were more likely to be infected compared to those earning Ghȼ 600 –1000 (AOR = 6.28; 95% CI: 1.58–11.94; *p* = 0.009). Moreover, residing in a compound house was associated with significantly increased odds of infection compared to living in a self‐contained house (AOR = 7.25; 95% CI: 1.23–12.43; *p* = 0.028). Regarding obstetric factors, secundigravidae had significantly higher odds of HBV infection than multigravida (AOR = 5.37; 95% CI: 2.45–11.80; *p* < 0.001). Women in their first trimester had substantially lower odds of infection than those in their third trimester (AOR = 0.02; 95% CI: 0.01–0.14; *p* < 0.001). Lastly, individuals with blood group O had significantly higher odds of HBV infection than those with blood group AB (AOR = 4.36; 95% CI: 1.40–6.54; *p* = 0.011).

**Table 4:** Sociodemographic and obstetric predictors of HBV infection among pregnant women.

		HBV status					
	Frequency	Positive	Negative	Model I		Model II	
Variable	*n* (%)	*n* (%)	*n* (%)	COR (95% CI)	*p* value	AOR (95% CI)	*p* value
Age							
18–25	174 (12.2)	3 (0.2)	171 (12.0)	1.51 (0.25–9.20)	0.150	0.54 (0.04–6.12)	0.126
26–30	1088 (76.1)	21 (1.5)	1067 (74.6)	1.63 (0.37–7.03)	0.210	1.02 (0.18–5.68)	0.180
31–40	168 (11.7)	2 (0.1)	166 (11.6)	1		1	
Marital status							
Not married	118 (8.3)	15 (1.1)	103 (7.2)	17.17 (7.68–24.34)	< 0.001*	17.51 (5.47–25.98)	< 0.001*
Married	1312 (91.8)	11 (0.8)	1301 (91.0)	1		1	
Educational attainment							
No formal education	414 (28.9)	20 (1.4)	394 (27.6)	2.09 (0.61–7.17)	0.218	0.50 (0.09–2.72)	0.108
Formal education	1016 (71.1)	6 (0.4)	1010 (70.7)	1		1	
Household structure							
Extended	1086 (75.9)	12 (0.8)	1074 (75.1)	0.26 (0.12–0.57)	0.001*	0.16 (0.05–0.52)	0.052
Nuclear	344 (24.1)	14 (1.0)	330 (23.1)	1		1	
Household type							
Compound house	1009 (70.6)	24 (1.7)	985 (68.9)	5.10 (1.20–7.69)	0.027*	7.25 (1.23–12.43)	0.028*
Self‐contain house	421 (29.4)	2 (0.1)	419 (29.3)	1		1	
Household category							
Mud with thatch	27 (1.9)	0 (0.0)	27 (1.9)	1		1	
Mud with iron sheets	129 (9.0)	3 (0.2)	126 (8.8)	1.29 (0.38–4.37)	0.177	1.66 (0.26–10.62)	0.128
Blocks with iron sheets	1274 (89.1)	23 (1.6)	1251 (87.5)	1		1	
Number of people in a household							
1–5	895 (62.6)	4 (0.3)	891 (62.3)	0.10 (0.03–0.30)	< 0.001*	1.11 (0.03–2.39)	0.101
6–10	532 (37.2)	22 (1.5)	510 (35.7)	1.23 (0.15–2.95)	0.224	0.83 (0.04–1.35)	0.208
11–15	3 (0.2)	0 (0.0)	3 (0.2)	1		1	
Gravidity							
Primigravida	335 (23.4)	4 (0.3)	331 (23.1)	1.41 (0.35–5.68)	0.128	3.70 (0.26–51.74)	0.330
Secundigravida	624 (43.6)	18 (1.3)	606 (42.4)	3.46 (1.16–10.31)	0.025*	5.37 (2.45–11.80)	< 0.001*
Multigravida	471 (32.9)	4 (0.3)	467 (32.7)	1		1	
Parity							
Multiparous	1134 (79.3)	17 (1.5)	1134 (77.8)	3.64 (1.02–12.93)	0.045*	0.20 (0.02–1.47)	0.340
Nulliparous	296 (20.7)	9 (0.3)	287 (20.4)	1		1	
Gestational age							
First trimester	1051 (73.5)	10 (0.7)	1041 (72.8)	0.22 (0.07–0.67)	0.008*	0.02 (0.01–0.14)	< 0.001*
Second trimester	256 (17.9)	11 (0.8)	245 (17.1)	1.05 (0.35–3.11)	0.216	0.11 (0.01–0.99)	0.059
Third trimester	123 (8.6)	5 (0.3)	118 (8.3)	1		1	
G6PD							
No defect	1371 (95.9)	11 (0.8)	1360 (95.1)	0.74 (0.28–0.93)	< 0.001*	0.53 (0.31–1.05)	0.054
Partial defect	49 (3.4)	12 (0.8)	37 (2.6)	0.75 (0.16–3.39)	0.116	0.13 (0.01–1.50)	0.104
Full defect	10 (0.7)	3 (0.2)	7 (0.5)	1		1	
Blood group							
O	337 (23.6)	13 (0.9)	324 (22.7)	4.19 (1.35–6.97)	0.013*	4.36 (1.40–6.54)	0.011*
A	422 (29.5)	4 (0.3)	418 (29.2)	2.55 (0.71–9.15)	0.149	2.51 (0.69–9.02)	0.158
B	420 (29.4)	3 (0.2)	417 (29.2)	0.75 (0.16–3.37)	0.110	0.75 (0.16–3.40)	0.117
AB	251 (17.6)	6 (0.4)	245 (17.1)	1		1	1

Abbreviations: %, percentage; ANC, antenatal care; AOR, adjusted odds ratio; COR, crude odds ratio; G6PD, glucose‐6‐phosphate dehydrogenase; HBV, hepatitis B virus; *n*, frequency.

* *p* < 0.05.

## 4 Discussion

### 4.1 Prevalence of HBV Infection

In the global fight against preventable maternal infections, understanding localized disease prevalence is pivotal for public health interventions. The overall prevalence of HBV among pregnant women in the Bono East Region was 1.82% (95% CI: 1.24–2.65). This is significantly lower than Ghana's national average of 8.36% [8] and far below figures reported in other regions of the country. For instance, earlier studies reported 4.2% [17] and 7.9% [18] in Northern Ghana, 4.4% in Suhum Municipality [19], and 6.0% in the Volta Region [20]. Outside of Ghana, higher rates have also been documented in other sub‐Saharan African settings, such as 6.0% in Nigeria [21], 6.3% in Kenya [22], and up to 11.2% in Somalia [23]. Nevertheless, our finding challenges the assumption of uniform HBV burden across endemic regions and stress the importance of context‐specific epidemiological surveillance to inform equitable health strategies. The differences in the rate of occurrence could be attributed to differences in geographical settings, differences in population mobility, maternal health access, socioeconomic factors, sociocultural practices, the effectiveness of antenatal interventions, and the strength of health policy.

A plausible explanation for the low prevalence rate Bono East Region could be the structured and continuous health education programs integrated into ANC services. In the selected study areas, there exist systematically crafted health promotional engagements that are routinely executed by trained health professionals. Such sessions may be less consistently applied in other regions. During these sessions, pregnant women are informed about various practices to enhance maternal and child safety. These include education on nutrition, lifestyle choices, risky behavior, early screening, and adherence to HBV vaccination, as confirmed by feedback during the IDIs and FGDs. For instance, it was emphasized that:

“…In our unit, the primary form of intervention is health education, and you have seen firsthand the thorough health education sessions we conducted this morning. As for HBV, because the vaccine is costly, pregnant women are educated about the harmful effects of HBV on both themselves and their infants, as well as the lifestyles that increase susceptibility to this infection. Subsequently, they are strongly encouraged to undergo HBV vaccination…”

(In‐charge, IDI‐KSD and IDI‐ATBM)

“…The nurses at the ANC sessions always provide us with extensive education on various diseases, including malaria, HIV, HBV, and UTIs. Thanks to their guidance, I now understand the preventive measures for HBV, such as avoiding the use of already‐used razor blades, condoms, and close contact with persons with HBV…”

(6th Pregnant woman, FGD‐NSM)

### 4.2 Behavioral Determinants of HBV Infection

Beyond the assessment of prevalence, this study examined the behavioral factors associated with increased odds of being infected with HBV. It was revealed that the risk of HBV infection was three times higher among pregnant women with a long‐standing tradition of communal sharing of sharp objects with other persons. This is consistent with reports from previous studies in Ethiopia [24] and Nigeria [25]. While these previous studies have linked HBV to shared personal items, our study uniquely situates this practice within household dynamics shaped by shared housing structures, offering a new contextual understanding of transmission pathways. The observation could be due to insufficient decontamination of items, which in turn leads to cross‐infection due to potential contact with infected fluids. This highlights the need for promoting safe personal hygiene practices among pregnant women and monitoring traditional practices such as scarification and circumcision.

Similarly, unprotected sex with sex partners was significantly associated with increased odds of HBV. This observation reinforces findings from earlier studies in Ethiopia, where unprotected heterosexual intercourse was associated with increased HBV seroprevalence [24, 26]. Contrary to these reports, our findings add another dimension of transmission by demonstrating this association within a predominantly married population. This suggests that relationship status alone may not confer protection and that health education must go beyond marital assumptions. One of the transmission routes of HBV is through semen and vaginal secretions [27]. Hence, transmission may surge with unprotected sexual activity, stressing the need to encourage protected sex and monogamous relationships.

In addition, pregnant women with a history of unsafe abortion were three times more likely to be infected with HBV, which agrees with a previous report [28]. In most African sub‐regions, the practices of induced abortion are mostly conducted in nonclinical settings with a poor culture of sterilization of instruments [29]. This presents a unique entry point for HBV transmission through contaminated instruments. Therefore, comprehensive post‐abortion care, including serological screening, vaccination, and treatment, could mitigate this risk factor.

Furthermore, blood transfusion was also another strong determinant of HBV transmission. This agrees with reports from an earlier study [28]. The other transmission routes of HBV include mucus and blood [27]. In the initial weeks post‐exposure, typically within the first month, HBV may not be detectable through standard diagnostic assays [30]. This phase is often followed by what is referred to as the “window period,” generally occurring between the fourth and twelfth week after infection [31]. During this time, serological markers such as HBsAg may not yet be present, despite active infection and the potential for onward transmission. As a standard practice, blood is thoroughly screened during pre‐donation to determine the presence of infection [32]. In an event where there are issues regarding the screening process, infected blood may be transfused to people, increasing the risk of infection. This highlights the need for mandatory HBV screening for all donated blood using sensitive nucleic acid testing and serological assays.

Moreover, our study is among the few in Ghana to quantify the association between informal cosmetic practices and HBV infection in pregnant women. Specifically, body piercing in nonclinical settings and the use of street nail trimming services emerged as significant risk factors. In Ghana, the widespread use of these practices reflects the country's rich tapestry of cultural norms and individual preferences. People adopt them for varied reasons, such as enhancing personal appearance, expressing cultural identity, or due to cost considerations. In the FGDs, some pregnant women expressed the following:

“…The abundance of earrings you see adorning my ears and nose is a distinct marker of my tribe's identity. In our culture, the more earrings one has, the more esteemed they are. We have individuals whose grandmothers have been specifically trained to pierce us whenever we desire. This practice has been an integral part of our cultural heritage…”

(3rd Pregnant woman, FGD‐TM)

Street‐based cosmetic services often operate without the infection prevention protocols found in clinical environments, including reliable sterilization practices, consistent hand hygiene, the use of antiseptic agents, and disposable equipment. This stresses the need for coordinated policies by health stakeholders to regulate these practices and to further improve the hygiene standards associated with street‐based cosmetic procedures.

### 4.3 Sociodemographic and Obstetric Predictors of HBV Infection

Our study revealed several significant sociodemographic and obstetric predictors of HBV infection among pregnant women in the Bono East Region of Ghana. Our findings have shed light on localized patterns that differ meaningfully from broader national and international trends. In this study, unmarried pregnant women were 17.51 times more likely to be infected with HBV, which is consistent with an earlier report [25]. Engaging in sexual relationships with multiple partners before pregnancy may elevate a woman's exposure to hepatitis B, particularly if any of those partners are asymptomatic carriers. In the absence of protective measures such as condom use, the risk of viral transmission is significantly heightened.

In addition, low‐income status emerged as a significant predictor. Pregnant women who earn lower monthly salaries have an increased likelihood of being infected with HBV. This observation corroborates an earlier report. Our observation mirrors findings from Somalia [33], where limited economic resources have been linked to increased exposure to health risks. This could be due to inadequate access to healthcare, low HBV vaccination uptake, and higher engagement in unsafe practices [33]. However, the income threshold in our study was substantially lower than in this setting. This indicates that even modest financial constraints in this region are likely to substantially increase HBV vulnerability. Notably, HBV vaccination and treatment come with a cost that each individual must incur. If diagnosed, individuals with low incomes may face challenges in accessing and affording HBV treatment, leading to a higher risk of associated complications. Equally, low earnings could intensify risky behaviors such as sharing items or unprotected sexual activities, increasing the risk of contracting HBV.

Living in compound houses was also associated with increased odds of HBV infection. In the context of this study, compound households are characterized by more individuals living in rooms separated by walls. This association is rarely highlighted in previous studies. To our knowledge, this is among the first studies in the Bono East Region to statistically establish housing structure as an independent risk factor for HBV in pregnant women. In such communal settings, increased interaction and sharing of personal items may facilitate horizontal transmission of HBV, especially when household norms support communal living with limited hygiene enforcement. For instance, household norms including sharing common amenities such as a kitchen, toilet, bathroom, and a common playground for children are likely to heighten poor hygiene and other risky behaviors, and these have been found to increase the risk of HBV infection [24].

Regarding gravidity, secundigravida were five times more likely to be infected with HBV, which is in line with an earlier study [34]. In secundigravid women, repeated contact with health facilities during antenatal, delivery, and postnatal care may increase the likelihood of hospital‐acquired infections, especially when exposed to invasive procedures such as blood transfusions, intravenous therapy, or surgery. Accordingly, the study also found a significant association between gestational age and HBV. This aligns with earlier findings from Northern Ghana [17, 18], suggesting cumulative exposure to healthcare environments and community‐level risks as pregnancy progresses. Finally, the study revealed that the odds of HBV infection were higher among pregnant women with O blood than among those with A, B, and AB blood. This observation was supported by a previous study [35].

### 4.4 Implications for Policy and Practice

The findings of this study highlight several critical policy implications that could drive meaningful changes in public health focus aimed at improving maternal health. First, universal HBV testing should be enforced at the first ANC visit, with a repeat screen in the third trimester for women with elevated risk profiles (such as secundigravidae, low‐income earners, residents of compound houses). Second, ANC platforms should systematically incorporate HBV‐related education, emphasizing routes of transmission such as sharing personal items and receiving cosmetic services in nonclinical settings. Also, public health campaigns should be focused on compound house residents, addressing shared living practices, safe personal hygiene, and myths surrounding HBV vaccination. In addition, authorities should consider developing guidelines and offering training for informal personal care providers (including street nail trimmers and traditional piercers) to improve infection control practices. Moreover, there should be an engagement with local leaders, traditional birth attendants, and peer educators to promote HBV screening and vaccination, especially among unmarried women and seasonal migrants who may face stigma or access barriers. Furthermore, stakeholders should introduce targeted subsidies or free vaccination schemes for pregnant women in low‐income brackets, particularly those unable to afford out‐of‐pocket costs for HBV immunization. Again, Regional health directorates should track HBV testing, vaccination status, and outcomes through digital health records, disaggregated by gravidity, housing, and income levels. Likewise, the Ministry of Health should reinforce adherence to national blood safety protocols and quality assurance in transfusion services, particularly in peripheral health facilities where screening capacity may be limited. Finally, there is a need to strengthen policies to ensure that children of HBsAg‐positive mothers receive both vaccination and hepatitis B immunoglobulin within the first 12–24 h of birth.

## 5 Conclusion and Future Research Directions

Amidst global efforts to eliminate the HBV, understanding local epidemiology among vulnerable populations remains a public health imperative. The study revealed a 1.82% prevalence of HBV among pregnant women, which was lower than the national prevalence of 8.36%. These striking differences suggest the presence of regional disparities potentially driven by contextual determinants such as local health education strategies, community norms, population mobility, and access to ANC. Our findings challenge the generalized assumption that HBV prevalence is uniformly high across all subnational regions in Ghana. Even though the prevalence was low, a series of factors were also significantly associated with increased odds of HBV infection among pregnant women. Despite the low prevalence of HBV among this vulnerable population, the study has contributed to a growing body of evidence that regional disparities exist and must be considered in health policy formulation. This localized approach is crucial for resource allocation, planning targeted interventions, and ensuring equity in health care access and outcomes. Our findings emphasize that lower prevalence should not diminish the importance of localized studies but rather reinforce the value of meticulous and context‐specific public health research. Continuous investigation and policy efforts are essential to ensure that all pregnant women receive the care and protection they need against HBV infection. Moving forward, future research should explore the effectiveness of expanded HBV vaccination programmes within antenatal settings, assess the integration of HBV prevention into maternal health education, and evaluate strategies for improving uptake of postnatal HBV prophylaxis, particularly among high‐risk subgroups. Longitudinal and implementation science studies would be instrumental in tracking the impact of such interventions over time.

### 5.1 Study's Strengths and Limitations

As a multicentre hospital‐based study, it provides a more holistic view of the burden of HBV among pregnant women by combining quantitative findings with qualitative perspectives. Despite these strengths, some limitations were inherent. First, the cross‐sectional nature of this study limits the causal inferences between the determinants and the occurrence of HBV infection. Furthermore, the use of self‐reported data for explanatory variables may introduce recall and social desirability bias. Also, the exclusion of pregnant women who did not attend ANC at the selected health facilities may underreport the actual burden of HBV among pregnant women in the region.

## Author Contributions

**Dennis Bardoe:** conceptualization, investigation, writing – original draft, methodology, validation, visualization, writing – review and editing, software, formal analysis, project administration, resources, data curation. **Daniel Hayford:** supervision, writing – review and editing, validation, methodology, investigation. **Robert Bagngmen Bio:** investigation, writing – review and editing, validation, methodology, supervision. **Denis Dekugmen Yar:** supervision, writing – review and editing.

## Conflicts of Interest

The authors declare no conflicts of interest.

## Transparency Statement

The lead author, Dennis Bardoe, affirms that this manuscript is an honest, accurate, and transparent account of the study being reported; that no important aspects of the study have been omitted; and that any discrepancies from the study as planned (and, if relevant, registered) have been explained.

## Data Availability

The data sets generated and/or analyzed during the current study are not publicly available due to anonymity and confidentiality, but are available from the corresponding author (Dennis Bardoe) at reasonable request. All authors have read and approved the final version of the manuscript. The lead author, Dennis Bardoe, had full access to all of the data in this study and takes complete responsibility for the integrity of the data and the accuracy of the data analysis.
